# Predicting future harm from gambling over a five-year period in a general population sample: a survival analysis

**DOI:** 10.1186/s12888-020-03016-x

**Published:** 2021-01-07

**Authors:** Shawn R. Currie, David C. Hodgins, Robert J. Williams, Kirsten Fiest

**Affiliations:** 1 Department of Psychology, University of Calgary, 2500 University Dr. N.W., Calgary, AB T2N 1N4 Canada; 2 Alberta Health Services – Calgary Zone, 10101 Southport Rd SW, Calgary, AB T2W 3N2 Canada; 3 University of Lethbridge Faculty of Health Sciences, 4401 University Drive, Lethbridge, Alberta T1K 3M4 Canada; 4 Department of Critical Care Medicine, University of Calgary, 3330 Hospital Drive NW, Calgary, Alberta T2N 4N1 Canada

**Keywords:** Low-risk gambling limits, Problem gambling, Modifiable risk factors, Longitudinal cohort study, Survival analysis

## Abstract

**Background:**

There is little longitudinal evidence on the cumulative risk of harm from gambling associated with excess spending and frequency of play. The present study sought to assess the risk of gambling problems over a five-year period in adults who exceed previously derived low-risk gambling limits compared to those who remain within the limits after controlling for other modifiable risk factors.

**Methods:**

Participants were adults (*N* = 4212) drawn from two independent Canadian longitudinal cohort studies who reported gambling in the past year and were free of problem gambling at time 1. Multivariate Cox regression was employed to assess the impact over time of gambling above low-risk gambling thresholds (frequency ≥ 8 times per month; expenditure ≥75CAD per month; percent of household income spent on gambling ≥1.7%) on developing moderate harm and problem gambling. Covariates included presence of a DSM5 addiction or mental health disorder at time 1, irrational gambling beliefs, number of stressful life events in past 12 months, number of game types played each year, and playing electronic gaming machines or casino games.

**Results:**

In both samples, exceeding the low-risk gambling limits at time 1 significantly increased the risk of moderate harm (defined as ≥2 consequences on the Problem Gambling Severity Index [PGSI]) within 5 years after controlling for other modifiable risk factors. Other significant predictors of harm were presence of a mental disorder at time 1, cognitive distortions about gambling, stressful life events, and playing electronic gaming machines or casino games. In one sample, the five-year cumulative survival rate for moderate harm among individuals who stayed below all the low-risk limits was 95% compared to 83% among gamblers who exceeded all limits. Each additional low-risk limit exceeded increased the cumulative probability of harm by 30%. Similar results were found in models when the outcome was problem gambling.

**Conclusions:**

Level of gambling involvement represents a highly modifiable risk factor for later harm. Staying below empirically derived safe gambling thresholds reduces the risk of harm over time.

## Background

Problem gambling is defined as “impaired control over gambling that results in significant harm for the gambler or people in his/her immediate social network” [1]. It remains a significant public health concern both in Canada [2] and internationally [3–5]. In the DSM 5, the term gambling disorder replaced the previous label of pathological gambling and the criteria were relaxed (committing illegal acts to finance gambling was removed as a criterion; the threshold of inclusion criteria was reduced from 5 of 10 to 4 of 9) [6]. Problem gambling is a generic term intended to encompass ‘gambling disorder,’ ‘compulsive gambling’, ‘addictive gambling’, etc. and continues to be the most common term used in the academic literature (since 2000). Population studies like the present investigation tend to favour the term problem gambling whereas studies involving treatment-seeking clinical samples favour the term gambling disorder. While problem gambling impacts approximately 1 to 3% of the general population [4], another 4 to 10% of the population are frequent gamblers (gambler at least twice weekly) or report symptoms of the disorder that lie beneath the clinical threshold [3]. Several comprehensive reviews of this literature [7–9] identify male gender, younger age, ethnic minority status, psychiatric comorbidity, family history of gambling disorder, lower socioeconomic status, and impulsivity as vulnerability factors for problem gambling. Among adolescents and young adults, the early risk factors include substance abuse, psychological distress, and poor academic performance [10].

There has been interest among researchers to study the relationship between gambling intensity and the development of problem gambling and associated harms. This research clearly shows that the more one gambles, the greater the likelihood of harm [11–13]. Gambling shares many of the same characteristics as alcohol in terms of the relationship between consumption levels and harm [14]. Like alcohol, gambling shows a reproducible dose-response relationship with increasing consumption leading to harm in most individuals [15–18]. This research is important for conceptualizing prevention opportunities because, unlike many of the established precursors of problem gambling, the amount one gambles is a modifiable risk factor. The concept of low-risk gambling limits, akin to the low-risk drinking guidelines, has been advanced by research teams in Canada [19], United States [20], and Australia [21]. Researchers have used cross-sectional survey data from gambling prevalence studies to identify the optimal frequency and expenditure thresholds for minimizing harm. A handful of studies have examined how exceeding low-risk gambling limits can predict the development of future harm [21, 22]. Canadian researchers showed that gamblers classified as low risk at time 1 (defined as gambling no more than three times per month and spending no more than 1000CAD per year on gambling) who shifted into high-risk gambling by a subsequent time point 14 months later were two to three times more likely to experience harm compared to gamblers who remained low risk at both assessments [22]. Australian researchers derived a set of low-risk limits from cross-sectional population data on gambling habits and then tested their longitudinal validity on a Tasmanian cohort of gamblers assessed across three time periods [21]. Exceeding the gambling frequency and expenditure limits at time 1 significantly predicted gambling-related harm in the subsequent time periods. The odds of experiencing gambling-related harm in subsequent waves increased by a factor of between 6 and 21 when these limits were exceeded.

Longitudinal studies of gambling behavior also provide the opportunity to study how various modifiable behavioural and clinical states interact and potentiate the risk of future problems over multiple time points. Canadian and US-based cohort studies reveal a pattern of relative stability of problem gambling at the mean level within samples; however there exists high inter and intra-individual variation over time. For example, in a Missouri-based study only one in 11 individuals classified as problem gamblers at the first cycle of data collection remained problem gamblers at the third cycle 7 years later [23]. One of the challenges with longitudinal studies is the narrow focus on problem gambling as the outcome of interest. Most prevalence studies find that problem gambling is present in less than 1% of the adult population. With the smaller samples that are typical of longitudinal investigations, only a small number of the gamblers followed over time would be expected to develop a disorder, and even fewer would retain this status across two or more time periods. There has been a growing interest in studying how harm manifests at sub-threshold levels [24]. Because problem gambling is typically proceeded by a period of at-risk gambling [8, 23], risk factors for developing at-risk levels of problem gambling can illuminate modifiable behaviours that can be targeted in prevention initiatives. Furthermore, research shows that although problem gamblers experience more harm and diminished quality of life at an individual level, most of the harm at a population level is accounted for by at-risk gamblers [25]. Statistically, at-risk or sub-clinical problem gamblers outnumber problem gamblers by a ratio of four to one [4].

Although several longitudinal investigations of gambling have emerged in the last 10 years, only a handful have used data from all time points to model the progression of gambling problems beyond point prevalence estimates at each assessment interval [26, 27]. Survival analysis is a statistical method that analyzes time to event outcome (survival) over multiple time points. In a basic survival analysis design, a cohort of individuals is measured on a single discrete outcome of interest (e.g., death, onset of disease, relapse, divorce) at multiple, equally spaced time points (weekly, monthly, yearly, etc.) over a fixed follow-up period. The main variable of interest is the time until failure—the point at which the outcome emerges.

The method has been adapted for addiction and mental health populations with outcomes such as time to depression relapse following treatment [28], onset of suicide ideation [29], development of alcohol use disorder [30], risk of hospitalization for psychiatric illness [31], relapse following treatment for gambling disorder [32] and time to recovery in gambling disorder [33]. A study with close relevance to the present investigation was a Danish cohort study that examined whether exceeding low-risk drinking limits increases the risk of hospitalization for psychiatric illness over the lifespan. Over 14,000 Danish adults were followed for up to 26 years to determine how alcohol consumption above the recommended national limits (14 drinks per week for women, 21 drinks per week for men) raised the risk of acquiring a mental disorder requiring hospitalization after controlling for other factors such as age, smoking, and income [31]. Women who exceeded the low-risk drinking limit increased their risk of anxiety disorders twofold. However, there was no elevated risk for men who exceeded the 21 drinks per week limit across controlling for other variables.

The data sources for the current investigation were Leisure, Lifestyle, and Lifecycle Project (LLLP) and Quinte Longitudinal Study (QLS). These population-based longitudinal studies were launched to comprehensively study the development of gambling problems over time and identify reliable risk factors. In addition to individual dispositional variables (vulnerability factors, cognitive distortions about gambling, etc.) first onset of gambling problems was strongly associated with more frequent and heavier gambling involvement at baseline [34, 35]. With regular follow-up assessments incorporated into both studies spanning a five-year period, the availability of these data provides a unique opportunity to use survival analysis to examine how gambling intensity and other modifiable risk factors at baseline predicts the development of gambling-related harms over multiple, future time points. We recently developed a set of low-risk gambling thresholds using the LLLP and QLS data sources [36], building on previous work using cross-sectional Canadian data sources [37]. The present study sought to understand how exceeding the low-risk limits impacts the emergence of harm over a 5-year period. The specific objectives of the study were:

1. Using survival analytic methods, model the risk of harm over time in gamblers who exceed low-risk gambling limits compared to gamblers who remain within the limits.
2. Identify other significant predictors that contribute to the first appearance of gambling harm and problem gambling.

## Methods

### Data sources

The Leisure, Lifestyle, and Lifecycle Project (LLLP), described in detail in other sources [22, 38, 39], was a prospective five-year panel study of 1808 adolescents and adults living in rural and urban Alberta. Briefly, data were collected over four waves (covering the years 2006 to 2011) on multiple factors theoretically linked to the etiology and natural progression of gambling habits. Random digit dialing (RDD) recruited participants from the general population in Alberta as well as a proportion of individuals (*n* = 524; 29%) who were likely to develop gambling problems during the longitudinal follow-up period (individuals who were above the 70th percentile in gambling expenditure or frequency based on national population data). Participants completed a battery of self-report and administered tests covering gambling, substance use, personality, intelligence, mental health, life events, and social environment. Fourteen months separated each period of data collection. The current study sample consisted of adults who reported gambling at time 1 and had valid data for gambling activity and harms for at least one post-baseline assessment (*N* = 780). The rate of attrition between the first and last waves of data collection was 24%.

The Quinte Longitudinal Study (QLS) was initiated at the same time as the LLLP [35]. It recruited 4123 Ontario adults from the Quinte Region in southeastern Ontario, Canada. The time frame (also 2006 to 2011), goals, and content of the baseline and follow-up assessments were very similar to the LLLP. Sampling was also done via RDD within the Quinte region. A similar proportion (26%) of adults with at-risk gambling characteristics (defined as spending at least 10 CAD per month on gambling in the past year or engaging in slot machines or horse racing) was recruited. Both studies employed a similar rate of remuneration for participants (QLS: 50CAD vs. LLLP: 75CAD for initial assessment). The rate of attrition between Time 1 and 5 in the QLS was 4%, much lower than the LLLP. Assessment intervals within the QLS were separated by 12 months. The study sample comprised 3054 adults who reported gambling at time 1 and had valid data for the measures of interest for at least one post-baseline assessment. Both studies used a combination of in-person and online data formats to collect the data used in the present analysis. Most of the gambling measures were collected using web surveys, a method thought to enhance the honesty of participant answers to sensitive questions such as gambling losses and psychosocial harms [40].

### Assessment of gambling activity

The QLS and LLLP used the same measures for assessing intensity and breadth of gambling habits and for gambling problems. The core questions derived from the Canadian Problem Gambling Index (CPGI) [41], a self-report survey designed to collect descriptive information on gambling habits in population studies. The CPGI collects detailed information on participant engagement with the most common games of chance available in Canada including: lottery tickets; instant win tickets; electronic gambling machines (EGM); casino table games; games of skill for money against other people (cards, pool, etc.); sports betting; horse or dog racing, and; other forms of gambling including online games. In terms of gambling expenditures, QLS and LLLP participants were asked to estimate over the past year the amount spent on each form of gambling in a typical month. The question wording conformed to the recommended standard for producing the most reliable estimate of actual expenditure [42]. The questions used in the present study asked people “Roughly how much money do you spend on [gambling type] in a typical month?” (‘spend’ means how much you are ahead or behind, or your net win or loss in an average month in the past 12 months). The question is repeated for all types of gambling reported by the participant in the past year, a method that is far superior than global estimates for all gambling activities.

The total expenditure on all forms of gambling was estimated by summing the expenditures for the individual gambling formats. Both self-reported losses and wins were considered in the calculation of total expenditure. Due to the presence of several extreme outliers, monthly expenditure was winsorized; values exceeding the 99th percentile for the distribution were replaced with the next lowest value (3700CAD per month). The percent of income spent on gambling was calculated by dividing the total expenditure for the month by the participant’s gross monthly household income (to a maximum of 100%).

Frequency of gambling was also assessed separately for each gambling format. The 7-point categorical scale [41] used in each study was converted to a quantitative scale to estimate number of gambling days each month. For the LLLP, the conversion factor was: 1–5 times/year = 0.25 days; 6–11 times/year = 0.5 days; 1 time/month = 1 day; 2–3 times/month = 2.5 days; once per week = 4 days; 2–6 times/week = 16 days, or; daily = 30 days. The conversion factor used in the QLS was: less than once a month = 0.5 days; once a month = 1 day; 2–3 times a month = 2.5 days; once a week = 4 days; 2–3 times a week = 10 days; 4 or more times a week = 16 days. Overall frequency of gambling was calculated by summing the frequency values for the individual gambling formats resulting in a value ranging from 0 to 30 times per month.

Gambling activity was assessed at each assessment interval. The measures of gambling intensity—frequency of gambling, amount spent per month in Canadian dollars, and expenditure on gambling as a proportion of family income—were converted into dichotomous variables by applying by the low-risk gambling limits established through previous research [36]. Gamblers who exceeded any of the low-risk limits at time 1 (frequency ≥ 8 times per month; expenditure ≥75CAD per month; percent of income ≥1.7%) were deemed to be gambling above the low-risk threshold. Although these three dimensions of gambling activity are correlated, each independently predicts harm from gambling [43]. Each participant was assessed on the total number of low-risk limits exceeded at time 1 (range 0 to 3).

### Outcome variables

Gambling related harm was the primary outcome of interest. All harms were assessed using the Problem Gambling Severity Index (PGSI), a nine-item scale from the CPGI that assesses consequences and behavioural symptoms of problem gambling in the past 12 months [41]. The PGSI has well-established psychometric properties [44]. At the time of these studies, there was no validated measure of gambling-related harm. Although the PGSI is a measure of problem gambling severity, it assesses harms as well as behavioural symptoms. Using the Problem Gambling Severity Index (PGSI) we defined two levels of harm, both scored dichotomously. Moderate level of harm was defined as reporting at least two consequences from the PGSI items addressing feeling guilty, betting more than one can afford, recognition of a problem, health problems, financial problems, being criticized by others, and borrowing money to gamble. This is the same definition of harm employed in several investigations on low-risk gambling limits [11, 12, 22]. In previous work we found this definition of harm to have the best psychometric properties (highest area under the curve, sensitivity and specificity values) compared to alternative harm definitions [43]. We also studied problem gambling as an outcome, defined as scoring five or higher on the full PGSI scale Although eight was the original cut-off for identifying problem gambling, research has shown that use of this cut-off has good correspondence to clinically assessed problem gamblers in treatment, but poor correspondence to clinically assessed problem gamblers in the general population [45, 46]. More recent studies indicate score of five or higher demonstrate high sensitivity, specificity, and overall classification accuracy in detecting problem gamblers compared to clinician assessments [45, 47, 48]. The PGSI was normed on a small group of treatment-seeking problem gamblers [41] who tend to have a more pervasive and severe set of problems compared to problem gamblers in the general population. Lowering the PGSI threshold to ≥5 has been shown to successfully capture both treatment-seeking and non-treatment seeking problem gamblers [45].

### Covariates

Because our results are intended to inform prevention initiatives, we only included modifiable risk factors in the modelling. Covariates were selected based on being a changeable behaviour or a treatable comorbidity and a strong relationship with problem gambling. For the latter criteria we selected variables that were shown to be predictors of future problem gambling in the multivariate model of etiology developed from the LLLP and QLS datasets [34, 35]. We also used the results of a recently completed meta-analysis of problem gambling risk factors [49]. Covariates included in the model consisted of the presence of a comorbid mental illness and substance use disorders (SUD), number of stressful life events in the past year, participation in continuous types of gambling (EGMs or casino table games), and number of gambling cognitive fallacies. To assess comorbidities, both studies included a structured diagnostic interview used extensively in population research [50] to assess the presence or absence of DSM-defined major depression, generalized anxiety disorder, panic disorder, obsessive-compulsive disorder, alcohol use disorder and drug dependence in the past 12 months. The Life Events Questionnaire (LEQ) [51] assessed the number of significant life events (e.g., loss of employment) that may have occurred in participants in the past 12 months. A total of 58 different life events across nine categories, including relationships, work, and finances are assessed by the LEQ. The total score provides a general measure of number of stressful events in the past year. The 10-item Gambling Fallacies Measure (GFM) [52] was used to assess common cognitive distortions about gambling such as misunderstanding the random nature of games and believing that one can win by using a system. Higher scores on the GFM reflect fewer cognitive gambling distortions.

In addition to the above time-fixed covariates, we also included in the Cox model two time-dependent variables that were measured at each wave. Because gambling behaviour was expected to vary over time, we included number of different game formats played at each time point (range 1 to 8) as a predictor. Playing EGMs or casino games elevates an individual’s risk of gambling problems [22, 53]. Therefore, playing EGMs or casino games between assessment intervals was another time-dependent covariate. Although internet gambling has also emerged as a high-risk gambling format [54], the proportion of the LLLP and QLS samples who reported engaging in internet forms of gambling was too small (< 5%) to warrant inclusion as a predictor in the models.

### Statistical analysis

Because of differences between the LLLP and QLS in both the assessment interval spacing (14 months vs. 12 months) and number of follow-up waves (4 vs. 5) it was not possible to merge the datasets for the Cox hazards models. Although sampling weights were available for the LLLP, the QLS had no weights therefore all analyses were conducted on unweighted data. Censored cases were managed the same way in both samples. Both datasets contained left and right-censored data on gambling harms. Because our interest was predicting the incidence of new gambling-related harm, participants who were assessed as problem gamblers at time 1 (left-censored data), were excluded from the samples. Right censored cases consisted of participants who dropped out and participants who remained free of harm at the last assessment wave. In keeping with standard procedures for survival analysis, right censored case were retained by coding dropouts with the last available data point. Missing data among the remaining variables was minimal (< 1% of cases).

Separate Cox proportional hazards models were run on the two outcome variables of interest: moderate harm, consisting of reporting two or more consequences from the PGSI, and new onset of problem gambling (PGSI score ≥ 5). Sequential models were run with the presence of a mental disorder, presence of a SUD, GFM total, LEQ total, EGM or casino game play at each time period, and number of different gambling formats played at each time period entered as a block first. The main predictor of interest, number of low-risk gambling limits exceeded at time 1 (range 0 to 3), was entered last to assess the unique importance of gambling above the recommended limits after controlled for other, modifiable risk factors. All analyses were conducted with SPSS Version 25. Assumptions of proportionality of hazards and non-linearity were tested for each model and were found to be within acceptable limits. Among the continuous covariates, LEQ scores displayed a modest positive skewness (1.76). Because transforming the variable did not significantly improve the distribution the analysis was conducted on the original data.

## Results

### Differences in study samples

Table 1 compares the demographic characteristics of the LLLP and QLS samples. As previously reported [36] the gambling characteristics of the two samples were very similar. The most notable differences in the samples were age and marital status. The LLLP sample was on average 6 years younger and had a much higher proportion of single individuals. The 12-month prevalence rates of mental health disorders was high in both samples—18 and 33% for the QLS and LLLP, respectively. Similarly, the proportions meeting diagnostic criteria for a substance use disorder was 8 and 11% in the QLS and LLLP samples, respectively. Forty-three percent of QLS participants were below all low-risk gambling limits at time 1 compared to 63% of the LLLP sample. Exceeding 1, 2, and 3 low-risk limits was observed in 23, 19, and 14% of QLS gamblers, respectively. Within the LLLP sample, 14, 16, and 6% of gamblers exceeded 1, 2, and 3 low-risk limits, respectively.

**Table 1:** Leisure, Lifestyle, and Lifecycle Project (LLLP) and Quinte Longitudinal Study (QLS): demographics and gambling characteristics at time 1

Variable	*N* (%) or Mean (SD)	
	QLS (*N* = 3432)	LLLP (*N* = 780)
Gender		
Male	1585 (46)	334 (43)
Female	1847 (54)	446 (57)
Marital		
Single	391 (11)	320 (41)
Married/common law	2484 (72)	382 (49)
Separated/divorced/widow	557 (17)	78 (10)
Ethnicity		
Caucasian	2972 (87)	710 (91)
Non-caucasian	460 (13)	70 (9)
Work		
Employed/Student	2306 (76)	565 (72)
Unemployed/retired/ disability	1126 (24)	215 (28)
Age		
Mean	45.9 (14.0)	40.0 (16.9)
Median	46	43
Baseline PGSI score^a^	.57 (1.0)	.44 (.9)
Gambling Fallacies Measure	7.1 (1.4)	6.8 (1.6)
Life Events Questionnaire	3.3 (3.0)	14.4 (6.3)
Gambling intensity at time 1		
Mean monthly net win/loss (SD)^b^	145.3 (350.9)	128.0 (524.2)
Median monthly net win/loss^b^	45	35
Different forms of gambling played (median)	3	2
Played EGM or casino games	34%	39%

*PGSI* Problem Gambling Severity Index

^a^Individuals with PGSI ≥5 excluded

^b^Canadian dollars

### Survival analysis

**Fig. 1:**
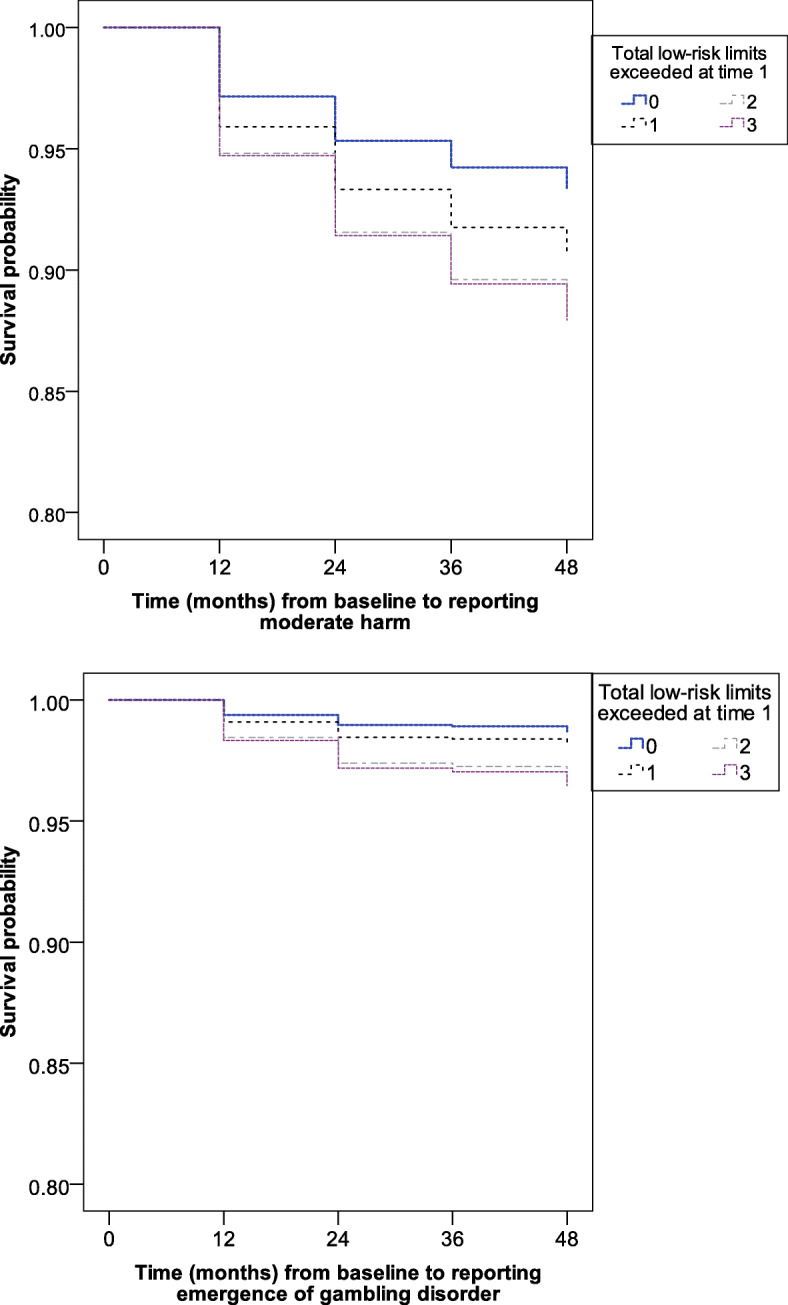
Predicted survival for the longitudinal outcomes of reporting two or more harms (top curve) and becoming a problem gambler (bottom curve) for the QLS dataset. Separate curves shown for gamblers who exceeded 0, 1, 2, or 3 of the low-risk gambling limits

The results of the Cox regression models are shown in Table 2. Using moderate harm as the outcome, the model on the QLS sample was highly significant before the addition of exceeding the low-risk gambling limits as a predictor (χ^2^ = 118.59; *p* < .001). The addition of number of low-risk limits exceeded as a predictor resulted in a significant change in the model χ^2^ value (12.59; *p* < .001). In the final model, mental health problems, stressful life events in the past year, number of game types played each year, playing EGMs or casino games, more cognitive distortions, and exceeding the low-risk limits all were independently predictive of future gambling harm. Each additional low-risk limit exceeded increased the cumulative probability of harm by 24%. Figure 1 displays the difference in the longitudinal survival proportions for gambles who exceeded 0, 1, 2, and all low-risk limits. The five-year cumulative survival rate for QLS gamblers who stayed below all low-risk limits was 95%. For gamblers who exceeded all the low-risk limits the cumulative survival rate was 83% at the last assessment.

**Table 2:** Cox proportional hazard model: Variables associated with developing harm from gambling over 5-year period

Variable	Longitudinal outcome variable			
	Moderate harm (≥ 2 harms)		Problem gambling (PGSI ≥5)	
	Hazards ratio (95% CI)	*p*-value	Hazards ratio (95% CI)	*p*-value
QLS sample				
Mental disorder	1.6 (1.2–2.2)	.003	1.9 (1.2–3.0)	.008
SUD	.90 (.6–1.4)	.590	1.7 (.90–3.0)	.099
Number of stressful life events	1.1 (1.0–1.1)	.024	1.0 (.9–1.6)	.320
Gambling fallacies	.9 (.81–.94)	.000	.8 (.7–.9)	.000
Number of gambling activities	1.2 (1.0–1.3)	.013	1.3 (1.1–1.6)	.009
Played EGMs or casino table games	1.8 (1.3–2.5)	.000	1.6 (.9–2.9)	.079
Low-risk gambling limits exceeded at time 1	1.3 (1.1–1.4)	.000	1.4 (1.2–1.7)	.000
LLLP sample				
Mental disorder	1.8 (1.2–2.6)	.002	2.2 (1.0–4.5)	.04
SUD	.8 (.4–1.5)	.472	1.3 (.5–3.7)	.64
Number of stressful life events	1.0 (1.0–1.1)	.043	1.0 (.9–1.1)	.47
Number of gambling fallacies	.9 (.8–.9)	.018	.7 (.6–.9)	.01
Number of gambling activities	1.2 (.96–1.4)	.123	1.3 (.96–1.9)	.082
Played EGMs or casino table games	1.3 (.88–2.0)	.313	4.0 (1.1–14.7)	.036
Low-risk gambling limits exceeded at time 1	1.3 (1.1–1.6)	.021	1.4 (.9–2.1)	.064

*EGM* electronic gaming machines, *SUD* substance use disorders

The same pattern of results was evident when problem gambling (PGSI ≥5) was used as the outcome. The model without exceeding the low-risk gambling limits was significant (χ^2^ = 107.07; *p* < .001). Adding the low-risk gambling limits predictor produced a significant change in the model χ^2^ value (11.58; *p* < .001). For every low-risk limit exceeded the probability of meeting criteria for problem gambling increased by 40%. In total, 54 gamblers become problem gamblers (1.9% of the baseline sample) during one of the four follow-up time points. As shown in Fig. 1, exceeding two low-risk limits had virtually the same survival pattern as exceeding three limits. At the end of the study period, the cumulative survival rate for avoiding problem gambling was 99% among individuals who adhered to all low-risk limits and 93% in gambling who exceeded all three limits.

Modelling conducted on the LLLP sample yielded similar results. The model predicting moderate harm was significant before the addition of exceeding the low-risk limits as a predictor (χ^2^ = 55.5, *p* < .001). The change in the model χ^2^ was significant (5.17; *p* < .05) after adding the low-risk limits factor. Significant predictors of moderate harm in the final model were experiencing a mental disorder, endorsing more cognitive distortions, more stressful live events, and exceeding the low-risk gambling limits. For each additional low-risk limit exceeded there was an increase in the cumulative probability of harm by 27%. The five-year cumulative survival rate among individuals who stayed below all the low-risk limits was 96% compared to 85% among gamblers who exceeded all limits.

**Fig. 2:**
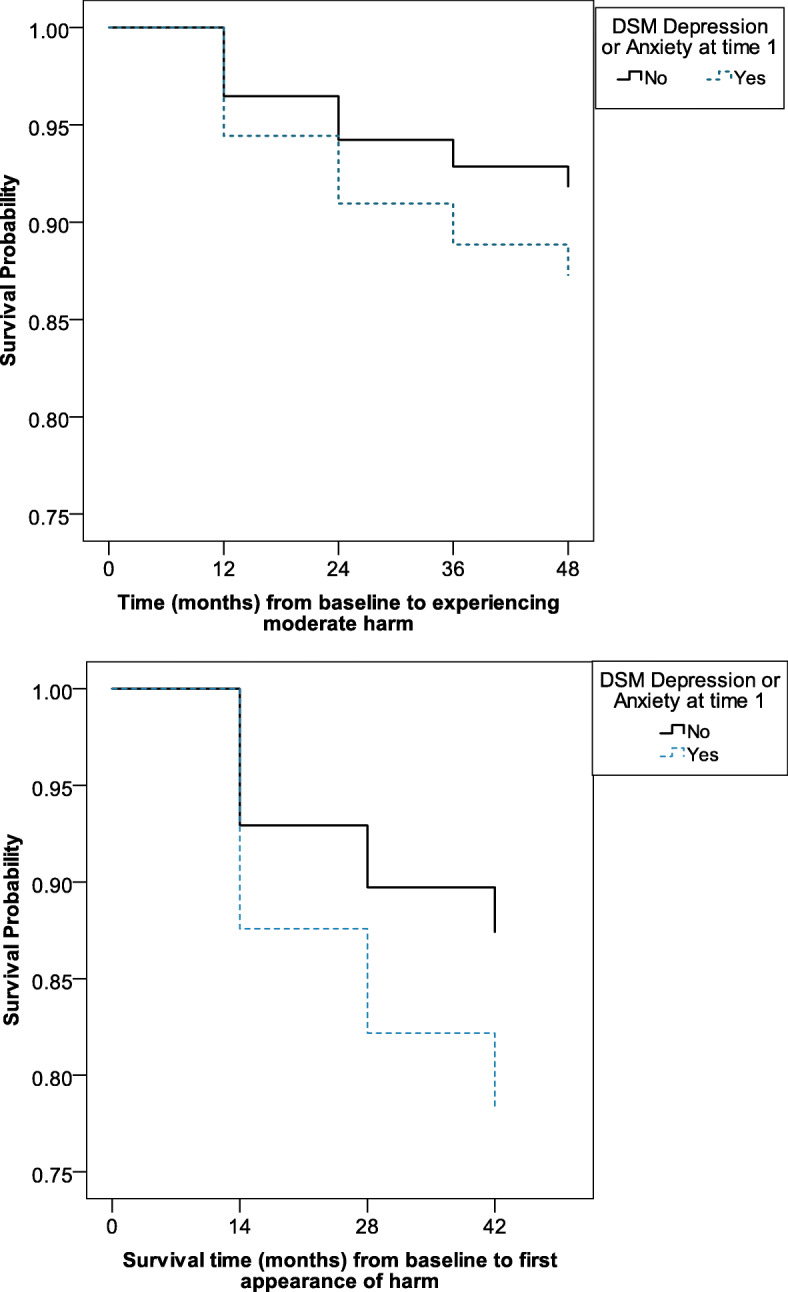
Impact of having a DSM-defined depression or anxiety disorder on avoiding moderate harm over a 5-year period in the LLLP (top) and QLS (bottom) study samples

Figure 2 illustrates how participants with an anxiety or depressive disorder at time 1 took less time to experience moderate problems from their gambling compared to persons without a mental disorder. The Cox regression model with problem gambling as the outcome measure did not show a significant incremental change in χ^2^ after adding the low-risk gambling limits factor (χ^2^ = 3.45; *p* > .05). In the overall model, the predictors of future harm were baseline mental illness, playing EGMs or casino games, and cognitive distortions. A smaller number of incident cases of problem gambling appeared in time periods 2 to 4 in the LLLP sample (*n* = 38; 4.9% of baseline sample).

## Discussion

The findings from two longitudinal cohort samples show that gamblers who exceed low-risk gambling thresholds for frequency, expenditure and percent of income spent on gambling are more likely to develop problems over a 5-year period compared to gamblers who remain within the limits, even after adjusting for other risk factors. For each low-risk limit exceeded, the probability of moderate harm increases by approximately 30%, and the probability of developing problem gambling increases by 40%. The overall pattern of results, including the significance of the other covariates tested in the models, was similar in both studies providing cross-validation of the predictors of future harm from two independent samples. Although we did not separately test the significance of exceeding 1, 2, or 3 low-risk limits the survival function plots suggest little differentiation between 2 and 3 low-risk limits—that is, gamblers who exceeded 2 low-risk limits had the same trajectory as gamblers who exceeded 3 limits. In addition to exceeding the low-risk gambling limits, future harm was also predicted by the presence of a mental disorder, stressful life events, number of gambling fallacies, and number of different gambling activities played each year. Common predictors of problem gambling in both samples were presence of a mental disorder, gambling fallacies, and number of different gambling activities played each year.

All the significant predictors represent modifiable behaviours or conditions. The mental health problems assessed in the LLLP and QLS consisted of depression and anxiety disorders, conditions for which there exists numerous evidence-based treatments. Prompt intervention for mental disorders could lower an individual’s risk for gambling problems. Stressful life events also predicted later gambling harm suggesting that distress levels below the threshold for a diagnosable mental disorder also increase one’s risk of gambling harm. Both of these findings could be useful in prevention initiatives intended to identify vulnerable populations who are at elevated risk for gambling problems.

The lack of significance for SUDs in the models was an interesting finding. There is a high rate of comorbidity between disorders of substance use and mental health; the presence of both covariates in the model resulted in only one achieving significance. Furthermore, the comorbidity of SUDs and problem gambling is well established however this finding is based largely on cross-sectional studies where the temporal relationship between conditions is unknown [5, 55, 56]. In the present analysis, individuals with problem gambling at time 1 were excluded. It appears that the presence of a SUD does not increase the cumulative risk of problem gambling after adjusting for other predictors.

Among the significant covariates were two related to other aspects of gambling. Number of different gambling formats provides an indication of the breadth of the individual’s involvement in gambling year after year. An increase in the number of gambling formats suggests a shift to more frequent and extensive gambling activity. Number of gambling fallacies reflects the cognitions and attitudes of gamblers. Other research has shown irrational gambling cognitions to be a strong correlate of problem gambling particularly in clinical samples [8, 57]. Previous research by the lead author also demonstrated that irrational gambling cognitions are positively related to both gambling intensity and symptoms of problem gambling at a population level [58]. Whereas most previous studies were conducted with cross-sectional data, the present longitudinal investigation shows that gambling fallacies are also predictive of later harm even after adjusting for baseline gambling intensity and other risk factors.

### Limitations

This study has several limitations that require acknowledgement. Foremost, the two study samples were drawn from specific areas of Canada. The results may not generalize to all provinces or to other countries. The higher attrition rate of the LLLP led to a smaller longitudinal sample and less robust results compared to the QLS. The diminished statistical power and fewer number of participants who developed serious problems by study end contributed to the lack of significance for low-risk limits within the model predicting problem gambling. We included in the Cox predictive models only a subset of variables that may be associated with gambling problems and did not include demographic factors. Our rationale was to focus on modifiable risk factors with variables pre-screened to have a strong correlation of gambling-related harms, with the intention to provide information useful for prevention efforts related to responsible gambling initiatives. Nonetheless, there may be several unmeasured factors that could have as much or even greater importance in shaping an individual’s gambling behaviours and risk for future harm. Although it has been subject to extensive validation studies [44, 45], the PGSI, is nonetheless a self-report screening measure for problem gambling. The PGSI is not a clinical tool and we have no means to confirm if all individuals scoring above our cut-off of five would be diagnosed with DSM-defined gambling disorder based on the gold standard of a clinical interview.

Exceeding the low-risk gambling limits significantly predicted both moderate harm and problem gambling, however the overall effect size in terms of the odds ratios and survival rates was small. In the predictive models, having a mental disorder had a stronger influence on future harm than the number of low-risk gambling limits exceeded. The oversampling of at-risk gamblers in both studies may account for this finding in addition to the high prevalence of mental disorders at baseline. For gamblers who exceeded all low-risk limits the five-year cumulative survival rate for avoiding moderate harm was 83%. When problem gambling was the outcome, the survival rate is 93%. Most gamblers who exceed the low-risk limits don’t develop problem gambling or experience moderate levels of harm. This is not an uncommon finding in predictive models involving health behaviours. Most individuals who exceed the low-risk drinking limits do not develop serious health consequences [59]. However, a much larger number of individuals who are moderate alcohol consumers experience harm compared to problem drinkers, a phenomenon that has been labelled the ‘prevention paradox’. Research conducted on a population samples in the United Kingdom and Australia has found evidence of the prevention paradox in gambling. Specifically, the total number of people who report harm at moderate levels of gambling far exceed the number of problem gamblers [25, 60]. Responsible gambling approaches that adopt a population strategy (e.g., public health campaigns that aim to correct erroneous believes about the odds of winning; promotion of safe spending limits) to reduce the average level of consumption in low to moderate risk gamblers are more likely to impact a larger portion of the population than approaches that target highly active, problem gamblers (e.g., casino self-exclusion programs, pop-up messages on EGMs that only appear after an hour of continuous play).

The low-risk gambling limits, like the Canadian low-risk limits for drinking, are intended to educate gamblers on the relative risk of exceeding certain thresholds. The comparison group is gamblers who remain below the limit (e.g., spend less than 75CAD per month) rather than individuals who abstain or gamble at very low levels. There is debate among researchers on the shape of the gambling dose-response curve with some advocating the true shape is linear or r-shaped suggesting that there is no basis for setting a discrete cut-off—any level of gambling above total abstinence is harmful [61]. In that case, an absolute risk approach might be better for setting a limit. With an absolute risk approach, a jurisdiction first must define how much risk would be considered acceptable to the general public. This is the approach taken by Australia for their version of the low-risk drinking limits. The drink thresholds are based on the risk of premature death from alcohol-related diseases being 1 in 100 or greater. A criticism of the absolute risk approach is that the determination of what is considered an acceptable level of risk is subjective. There are no standards for setting absolute risk levels for addictive behaviours [62, 63]. It also appears the public has a higher tolerance for voluntary behaviours such as drinking and gambling, than involuntary risks such as exposure to radiation or contaminants in air or water supplies [64] where the typical absolute risk threshold is 1 in a million.

The present study should help to inform the expansion of responsible gambling advice to include quantitative thresholds. Canada’s work in developing low-risk gambling limits has since expanded to include many international collaborative partners who have expressed the desire to adopt the limits for their own country [14]. With endorsement by multiple stakeholders including treatment providers, responsible gambling advocacy groups, and government regulators, low-risk gambling limits will eventually enjoy broad circulation with other public health initiatives.

## Data Availability

Please contact the Gambling Research Exchange Ontario to access the data.

## Abbreviations

CPGI: Canadian Problem Gambling Index
EGM: Electronic gaming machines
GFM: Gambling Fallacies Measure
LEQ: Life Events Questionnaire
LLLP: Leisure, Lifestyle, and Lifecycle Project
PGSI: Problem Gambling Severity Index
PG: Problem gambling
QLS: Quinte Longitudinal Study
RDD: Random digit dialing
SUD: Substance use disorders
